# Provider responses to discontinuous tariffs: evidence from Dutch rehabilitation care

**DOI:** 10.1007/s10754-021-09322-5

**Published:** 2022-02-01

**Authors:** Katalin Gaspar, Xander Koolman

**Affiliations:** School of Business and Economics, Section Health Economics, Talma Institute/VU University Amsterdam, De Boelelaan 1085, 1081 HV Amsterdam, The Netherlands

**Keywords:** Provider payment, Provider incentive, Policy evaluation, Regulated competition, The Netherlands, I11, I13, I18

## Abstract

Abrupt jumps in reimbursement tariffs have been shown to lead to unintended effects in physicians’ behavior. A sudden change in tariffs at a pre-defined point in the treatment can incentivize health care providers to prolong treatment to reach the higher tariff, and then to discharge patients once the higher tariff is reached. The Dutch reimbursement schedule in hospital rehabilitation care follows a two-threshold stepwise-function based on treatment duration. We investigated the prevalence of strategic discharges around the first threshold and assessed whether their share varies by provider type. Our findings suggest moderate response to incentives by traditional care providers (general and academic hospitals, rehabilitation centers and multicategorical providers), and strong response by profit-oriented independent treatment centers. When examining the variation in response based on the financial position of the organization, we found a higher probability of manipulation among providers in financial distress. Our findings provide multiple insights and possible indicators to identify provider types that may be more prone to strategic behavior.

## Introduction

Tariff schedules in healthcare often follow step-functions, where the tariffs for treatments increase (or decrease) based on certain criteria (e.g. treatment duration) having been met. Due to the inherent financial incentives, sharp discontinuities in reimbursement schedules may cause distortions away from the optimal treatment. A provider might, for example, extend treatment duration beyond what is medically necessary in order to qualify for a higher tariff and discharge patients directly after the threshold. Or conversely, a provider might cut the treatment short once a higher tariff has been reached and no further incremental increase in tariffs is obtainable.

Such sharp discontinuities have been used to assess providers’ responses to financial incentives in healthcare. Two often-cited papers on Medicare’s prospective payment system for long-term acute-care (LTAC) facilities have shown that providers respond to a large, one-off increase in tariffs by delaying hospital discharges until the higher tariff is reached and discharge a large proportion of patients directly after (Einav et al., 2018; Eliason et al., 2018). A similar study was conducted by Douven et al. (2015) in Dutch mental health care who contrasted self-employed providers with budgeted organizations, and observed spikes in the distribution of treatment durations at or directly after a rise in reimbursements for self-employed providers, but found no response for budgeted hospitals. Sharp discontinuities in tariffs have also been shown to lead to “upcoding” of birthweights in German neonatal units (Jürges & Köberlein, 2015; Reif et al., 2018).^1^^,^^2^

Even though there is growing amount of evidence indicating strong response to financial incentives by, at least, some healthcare providers, we still do not sufficiently understand the exact drivers of this behavior. The goal of our paper is to add to this literature by assessing the magnitude of response by medical rehabilitation providers in the Netherlands. Our findings suggest a moderate response by traditional care providers (general and academic hospitals, rehabilitation centers and multicategorical providers), and strong response by profit-oriented independent treatment centers.

The financial health of stand-alone rehabilitation care providers (e.g. specialized rehabilitation centers, and independent treatment centers) has been badly hit in recent years due to a combination of factors (Skipr, 2019; Wilman, 2020). There is ample anecdotal evidence indicating that the weak financial position of certain organizations might induce manipulation of treatment times in order to optimize profits and potentially ‘save’ the organization from bankruptcy. In addition, Silverman & Skinner, 2004 found that hospitals in financial distress were more likely to “upcode” in the United States (Silverman & Skinner, 2004). Therefore, in the second part of the paper, we test whether there is statistical evidence to prove that rehabilitation providers in financial distress were more likely to manipulate than financially healthy providers.

Much of the research mentioned above builds on the so-called bunching approach. A fairly recent development in empirical economics, which uses bunching around discontinuities in financial incentives to elicit behavioral responses by agents and estimate structural parameters. (Kleven, 2016) However, our conceptual framework is closest to the work of Eliason et al., 2018 according to which the provider considers at each period whether or not to end the treatment of a patient by weighing the pros (i.e. additional benefit to patient, additional revenue for provider) versus the cons (i.e. additional costs associated with extra treatment, risks incurred by discharging patient). Therefore, rather than fitting a curve to the distribution of discharges by treatment hour in order to find the counterfactual curve as suggested by Kleven (2016), we constructed our dataset by treatment period and evaluated the probability of a treatment ending at each period for each patient.

Based on our results we have concluded that response to financial incentives is not universally present among all providers, with traditional care providers [general hospitals (GH) and university medical centers (UMCs), rehabilitation centers (RCs)] showing no, or only moderate response, while profit-oriented independent treatment centers (ITCs) showing strong response. Furthermore, we found that providers in financial distress were considerably more likely to utilize strategic discharge than financially health organizations.

We used claims-level data provided by one of the largest health insurers in the Netherlands [Centraal Ziekenfonds (CZ)], representing 21% of the country’s insured (Vektis Intelligence, 2018). We built on the assumption that providers in the Netherlands treat patients independently of which private health insurer they are insured with. This assumption, albeit to our knowledge unproven, seems reasonable given the unique structure of Dutch health care system: nearly all residents of the Netherlands are insured by one of the several private health insurers and practically all secondary care (including rehabilitation care) is included in the basic package provided by all health insurers (Kroneman et al., 2016). Therefore, we consider our findings using one insurer to be indicative of the national trends.

The outline of our paper is as follows. In Sect. 2, we provide a brief overview of the Dutch hospital care with a focus on rehabilitation care. In Sects. 3 and 4, we describe our data and our estimation methods. In Sect. 5, we present our results and in Sect. 6 we discuss and conclude.

## Overview of rehabilitation care in the Netherlands

Rehabilitation care providers work with patients with various neurological, musculoskeletal, orthopedic and other medical conditions following stabilization of their acute medical issues. A long list of acute conditions may require rehabilitation care including injuries and trauma, stroke, major surgeries, birth defects, developmental issues, or chronic pain due to other sources (Krug & Cieza, 2019; Rauch et al., 2019). Due to the wide range of conditions and the highly individualized needs of patients, treatment tends to be multi-disciplinary including medical specialists, physical therapists, speech therapists, occupational therapists and mental health care providers. Depending on the severity of the damage and the type of care required, rehabilitation care may be conducted in an inpatient or outpatient setting.

Rehabilitation care in the Netherlands is primarily provided by GHs, UMCs, specialized rehabilitation centers (RCs), and ITCs, and a minority are provided by multi-categorical providers. In general, RCs treat the most severe patients mainly in the subcategories of brain disorders, amputations and paraplegia, in outpatient as well as inpatient settings. GHs and UMCs provide care to the general population of patients not critical enough for RCs and cover nearly all subcategories but solely in a non-clinical setting. ITCs treat less complex routine cases mainly in the subcategories of chronic pain and mental disorder and musculoskeletal disease.^3^ Furthermore, a small fraction of the claims is registered by multi-categorical providers that provide rehabilitation care in addition to other types of care, such as mental health care, and by independent physicians.^4^

Before visiting a rehabilitation care provider, patients must obtain a referral from their general practitioner (GP).^5^ Although referrals do not restrict the patient’s choice of provider, GPs generally indicate providers in the area that are appropriate for the patient’s condition and urgency of care. After the initial visit, the provider has 9 weighted hours of contact to establish a diagnosis, setup a treatment plan, discharge the patient entirely, or to transfer the patient to another (type of) facility.^6^ During this period, for instance, patients may be transferred from an ITC to an RC, depending on medical needs.^7^

Demand for rehabilitation care has been steadily growing in recent years, leading to waiting lists at most rehabilitation facilities. According to government regulations, the provider has 4 weeks after initial contact to organize a first visit with a physician (Kroneman et al., 2016). In 2016, the average national waiting time in outpatient care was 4.9 weeks for traditional providers, albeit slightly shorter for ITCs (Revalidatie Nederland, 2017). However, the financial performance of the sector has been weak due to a combination of factors: outdated tariff prices, a shift from inpatient to outpatient care that led to revenue losses for some providers, an increasing use of intensive treatments, and a shortening of the inpatient hospital stays (Skipr, 2014). As a consequence, higher patient turnovers were combined with weaker financial figures. In 2018, at least half of RCs facilities made losses, and set off a trend of consolidation within the industry (Skipr, 2019).

### Institutional background

Health care in the Netherlands is built on the fundamentals of regulated competition, a system in which residents are required by law to purchase basic health insurance from a selection of private insurers. These insurers are trusted with care procurement included in the compulsory insurance package for all their plan-holders. Health insurers generally have contracts with all large institutions: GHs, UMCs and specialized care facilities (e.g. rehabilitation and dialysis centers) for services included in the basic package and potentially more. In addition, insurers may contract selected ITCs. Patients are reimbursed fully above the yearly front-end deductible amount (between €350–385 during period of our study) for all rehabilitation care at contracted providers (in-network). In addition, they are partially reimbursed (generally between 70 and 80% of the full amount) for care consumed at non-contracted (out-of-network) providers (Kuijper, 2018). Staff employed in rehabilitation care (e.g. physicians, physiotherapists, occupational therapists) are almost exclusively paid on monthly fixed salaries.^8^ Health care organizations are non-profit private institutions. Profit distribution to investors remains prohibited for all inpatient facilities (i.e. all GHs, UMCs, RC), but it is permitted for outpatient facilities (mainly ITCs) (SiRM en Finance Ideas onderzoek naar uitkeren van dividend in de zorg naar Tweede Kamer gestuurd, 2019).

## Data

Rehabilitation care in the Netherlands is categorized into 7 types of care and 1 additional categorized as short rehabilitation. (See Table 1 for list of subcategories). Majority of care (97% of claims in our dataset) is performed in outpatient facilities, this segment is the focus of our analysis. 65% of the care is categorized as short rehabilitation with a maximum weighted treatment hours (WHR) of 9 h (referred to as DRG0 in our paper). This period is devoted to establishing a diagnosis and setting up a treatment plan. During this period a large share of patients are transferred to other types of rehabilitation facilities (e.g. more complex cases at ITCs may be transferred to RC, or vice versa, cases deemed less complex may be transferred from RCs to ITCs. In addition, patient may be transferred to geriatric rehabilitation.). Once the treatment duration crosses the 9 weighted hour mark, a diagnosis and subcategory of care is established and treatment begins. In order to avoid a large amount of transfers to other (types of) rehabilitation care biasing our results, we leave short rehabilitation out of our statistical analysis. While the thresholds varied between subcategories, the logic remains similar for all with 3 DRG levels (i.e. 2 thresholds and 3 tariffs) based on the weighted length of treatment. (See Table 5 in the Appendix for exact thresholds and see Table 6 in the Appendix for average prices per DRG).

**Table 1:** Number of claims per provider type and subcategory

Subcategories	GHs + UMCs	ITCs	RCs	Other	Total
1. Brain disorder	3007 (3.35)	314 (1.56)	16,513 (13.27)	1521 (10.98)	21,355
2. Organ disorder	1030 (1.15)	152 (0.75)	4268 (3.43)	301 (2.17)	5751
3. Musculoskeletal system disorder	1655 (1.84)	1604 (7.95)	4,404 (3.54)	611 (4.41)	8274
4. Nervous system disorder	1665 (1.85)	159 (0.79)	5674 (4.56)	457 (3.30)	7955
5. Amputation	272 (0.30)	5 (0.02)	786 (0.63)	109 (0.79)	1172
6. Chronic Pain and mental disorders	2056 (2.29)	2000 (9.92)	7950 (6.39)	501 (3.62)	12,507
Subtotal	9685 (10.78)	4234 (20.99)	39,595 (31.82)	3,500 (25.27)	57,014
7. Short rehabilitation	80,085 (89.10)	15,910 (78.88)	83,554 (67.14)	10,103 (72.92)	189,652
Total	89,770 (100)	20,144 (100)	123,149 (100)	13,603 (100)	246,666

Percentage of total claims (%) in parentheses

### Claims data

Claim level data was provided by CZ. The dataset comprises all claims registered in hospital rehabilitation care for the period of 2015 and 2018. A claim was opened for every patient visiting a health facility. The maximum opening time for a claim is 90 days; after which it was automatically closed and processed. Our dataset was restricted to claims submitted by contracted parties of the health insurer where medical activities were available. Identification codes for individual patients and for health care provider had been anonymized by the provider of the data.

### Treatment duration and weighting

Each claim contained information on the type of medical activity performed during the treatment (e.g. physician contact, physiotherapy, occupational therapy, psychological therapy). Each medical activity recorded was worth 5 min of unweighted treatment time and was multiplied by the weighting multiplier for the type of activity to obtain the weighted treatment time. For example a 5-min contact with a physician equaled 14.5 min of weighted treatment time using the multiplier for physicians of 2.9, while the same amount of time with a physiotherapist was worth 5 min using a multiplier of 1.

### Provider types

We categorized providers into 4 groups (1. GHs and UMCs, 2. RCs and 3. ITCs 4. Other) based on official hospital registration codes (General Data Management Codes).^9^ There were in total 69 in-network rehabilitation care providers in our dataset: 36 general hospitals and UMCs, 20 rehabilitation centers, 10 ITCs and 3 other providers.

### Financial position of the provider

The financial position of the provider was proxied using their net profit margins (NPM). Organizations with NPM < 0 were categorized as in financial distress.^10^^,^^11^

## Methods

### Analysis I: evidence of manipulation

In Fig. 2 in the Appendix, we present the distribution of WHR per provider type indicating strong visual evidence of manipulation in the case of certain providers. The figure also shows that the manipulation primarily occurs around the first threshold (T1) (where claims move from DRG1 to DRG2), while the number of claims closed later in the treatment process decreases quickly, leading to a long and narrow tail. We, therefore, re-centered WHR around the T1, making the first hour of care in DRG2 equal to zero.

As per Eliason et al., 2018 we assumed that providers consider whether or not to discharge the patient periodically throughout the treatment process (e.g. after every hour of treatment). Therefore, we created a new dataset based on these periods per patient and created a binary variable “Treat_End” equal to zero if treatment continued and equal to 1 if patient was discharged. We combined all subcategories of care per provider and ran a series of probit regressions estimating the probability of a treatment ending at each period in relation to the threshold. However, as subcategories of care have different ranges of treatment times, we needed to first create an index of treatment times ‘t’ where the distance from the beginning of the treatment to the first threshold is normalized for all subcategories.^12^^,^^13^ We estimated the following model:

$$ \Pr \left( {Treat\_End{|}t, t_{before} , t_{at} } \right) = {\Phi }\left( {\beta_{0} + \beta_{1} t + \beta_{2} t^{2} + \mu_{1} t_{before} + \mu_{2} t_{at} } \right) $$

where t is the duration of treatment using our normalized index, $t_{before}$ and $t_{at}$ indicating dummy-variables equal to 1 when treatment duration is directly before and at the threshold, respectively. We assumed that the counterfactual distribution of total treatment times without the disturbances caused by manipulation would have been monotone decreasing and convex.^14^ We modeled this trend by adding the quadratic term t^2^ to our regression.

According to the bunching literature, we expected an excess mass of discharges at the threshold, and hole (or missing mass) directly before the threshold (Kleven, 2016). These deviations from the counterfactual trend were captured by the two coefficients ${\mu }_{1}$ and ${\mu }_{2}$, but it was their relative value rather than their absolute magnitude that proved the presence of manipulation.^15^ More specifically, we expected the probability of discharge at the threshold to be higher than directly before. We captured the relative value ${\mu }_{1}$ and ${\mu }_{2}$ by their ratio, referred to as the *probability ratio*. A probability ratio above 1 indicated a relative increase at ${\mu }_{2}$ compared to ${\mu }_{1}$ and a presence of manipulation. We used the Wald-test to test that the ratio is statistically different from 1.

### Analysis II: estimating the effect of providers’ financial position

In this section, we examined whether financial distress of the provider is associated with more discharge manipulation. We divided our dataset into subgroups based on the provider’s level of financial health (1. financially healthy, 2. in financial distress) and, similarly to the methods outlined above, calculated their probability ratios. As higher use of discharge manipulation in one group led to higher probability ratio, similarly to Analysis I, we compared these two probability ratios by creating a ratio of the probability-ratios and using a Wald-test to test whether they significantly different from 1.

## Results

### Descriptive statistics

Our final dataset contained about 57 thousand claims for patients registered in 68 rehabilitation facilities. Table 1 presents the distribution of the dataset by subcategory and provider type. RCs produced the largest number of claims in all subcategories, but their share was particularly high in the subcategory of brain disorders. Conversely, the share of ITCs was highest in the subcategories of chronic pain and musculoskeletal system disorders.

Table 2 contains summary statistics for all providers and subcategories excluding short rehabilitation. Considerable variation was shown in patient characteristics per provider type. The share of patients with any kind of comorbidities registered in the previous year was between 44 to 72% with ITCs treating the population of patients with the lowest share with comorbidities (44%) and RCs treating patients with the highest share (74%). Nonetheless, ITCs registered the highest average weighted treatment duration of 38.6 h (compared to 31.65 h for RCs), but with relatively low average reimbursed tariffs of €3,407 (compared to €3,831). (See summary statistics per subcategory of care in Table 6 of the Appendix).

**Table 2:** Summary statistics (excluding short rehabilitation)

	GH + UMC	ITC	RC	Other
Mean weighted hours of treatment	21.76	38.93	31.65	31.10
SD weighted hours of treatment	13.96	34.88	24.92	26.36
Female (%)	54.83	64.61	49.99	49.82
Mean age (years)	45.00	49.14	36.08	35.76
SD age (years)	20.96	14.89	23.66	23.61
Presence of other comorbidities (%)^a^	70.02	44.32	71.61	66.68
Mean reimbursement per claim (€)	2985	3421	3832	3902
SD reimbursement per claim (€)	1241	1748	2438	2624
Number of providers (#)	36	9	20	3
Number of providers without financial information	5	0	6	0
Share of claims without financial information (%)	12.99	0	49.63	0
Number of providers in financial distress (#)	6	7	7	0
Share of claims from provider in financial distress (% of total claims)	9.94	55.30	29.59	0

SD stands for standard deviation

^a^Based on primary diagnosis-cost groups (‘Diagnose Kosten Groepen’ or DKG codes) with diagnosis in the year prior to rehabilitation treatment

**Fig. 1:**
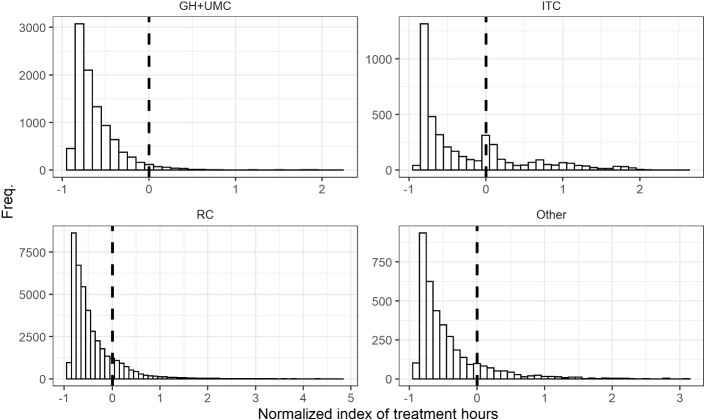
Distribution by provider type by normalized index of treatment hours (centered at T1)

**Fig. 2:**
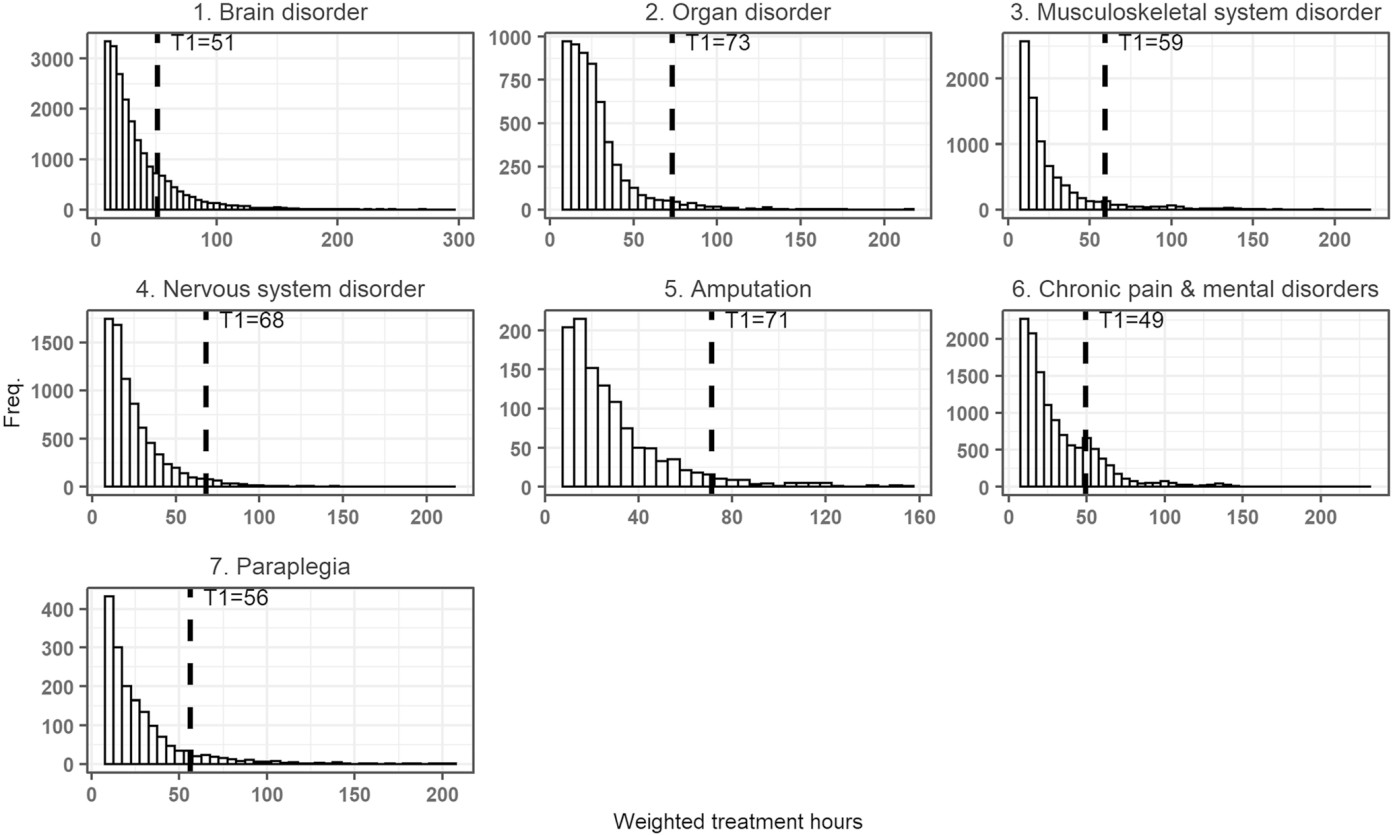
Weighted treatment times by subcategory

**Fig. 3:**
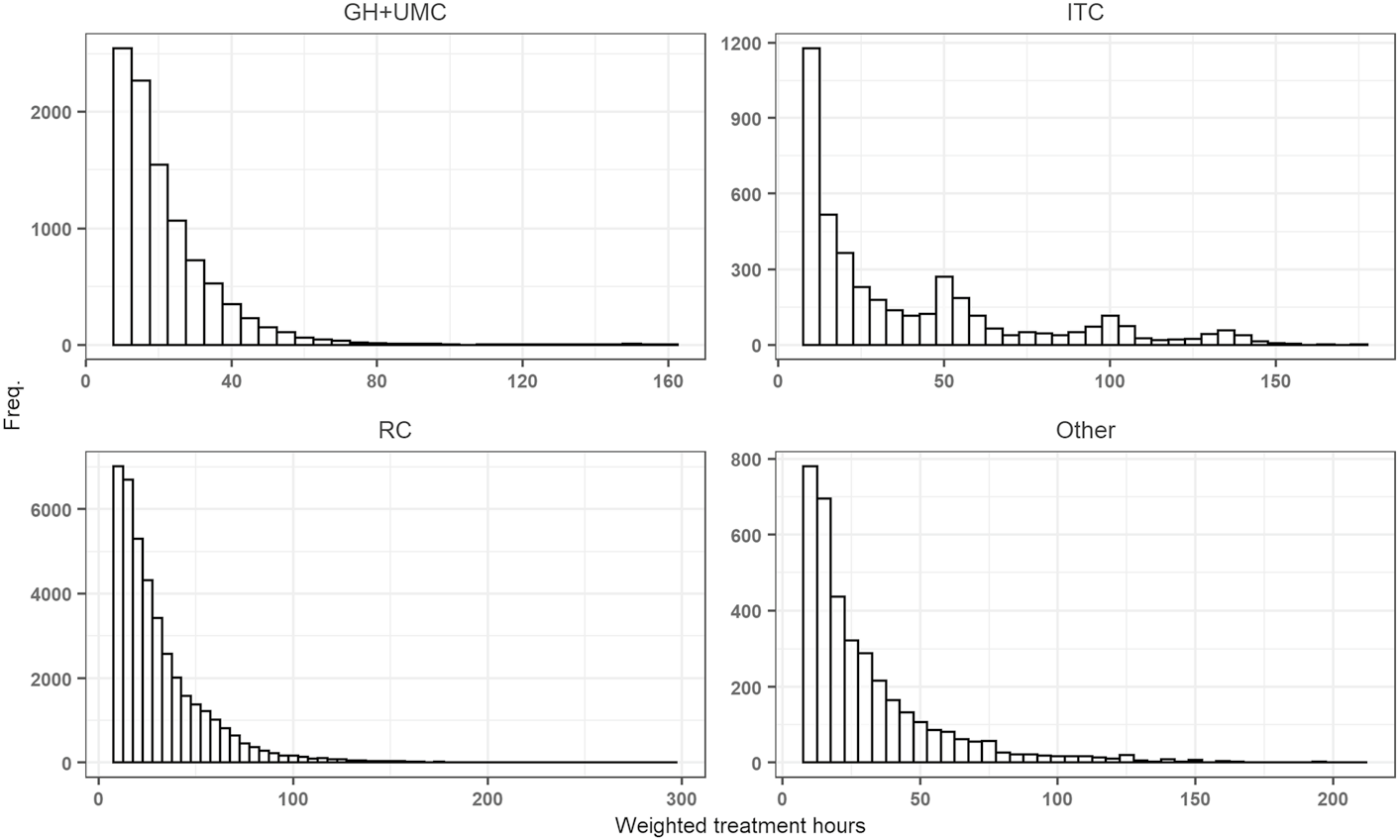
Weighted treatment hours by provider type

In Fig. 1 we present the distribution of discharges using our normalized index of treatment times by provider type. Using simple visual inspection, we concluded that three of the four curves depict a convex shaped decline without any bunching, while for ITCs significant bunching is visible at the threshold. In Figs. 2 and 3 in the Appendix, we present distributions by un-centered and un-normalized treatment hours by subcategory (aggregated for all providers) and by provider type (aggregated for all subcategories). Figure 3 in the Appendix indicates bunching in the subcategory of chronic pain and mental disorders.

### Analysis I

Table 3 depicts the discharge probabilities at depicts the discharge probabilities at our two main points of interest (before and at the threshold) and their ratios, in order to illustrate the magnitude of the *relative* change. The probability of discharge was somewhat larger at the threshold than directly prior for all provider types, although this difference was only modest for GH and UMCs, RC and the other category. However, there was a considerably large difference for ITCs. The probability ratio (depicted in the last column) was 3.65 for ITCs compared to 1.097, 1.056 and 1.393 for GH and UMCs, RC and the other category, respectively.

**Table 3:** Probability of discharge at the threshold and directly before the threshold

Provider types	Probability at threshold	Probability directly before threshold	Probability ratio^a^
GH + UMC (N = 209,952)	0.085	0.078	1.097***
	(0.005)	(0.004)	
ITC (N = 163,699)	0.156	0.043	3.649***
	(0.005)	(0.003)	
RC (N = 1,248,089)	0.086	0.082	1.056***
	(0.001)	(0.001)	
Other (N = 108,940)	0.092	0.066	1.393***
	(0.005)	(0.004)	

Standard errors in parentheses. N stands for number of observations

*p < 0.1; **p < 0.05; ***p < 0.01

^a^Probability ratios are bootstrapped in order to obtain confidence intervals. *P* values for Wald-test for H_0_: Probability Ratio = $\frac{{{\text{Probability}}\,{\text{ at}}\,{\text{ threshold}}}}{{{\text{Probability}}\,{\text{directly}}\,{\text{before}}\,{\text{threshold}}}} = 1$

### Analysis II

In this subsection, we re-estimate the probit-regression to test whether the probability of a strategic discharge was higher for providers that were in financial distress. Table 4 presents the marginal effects for each subgroup of analysis. The probability of discharge directly before the threshold and at the threshold were similar for GHs and UMCs in both subgroups, leading to probability ratios close to 1. On the other hand, strong difference in the probability ratios was found for ITCs, where a 4.8-fold difference was observed in the probability of a discharge at the threshold versus before the threshold for organizations in financial distress, while only a 1.5-fold difference was observed for financially healthy organizations. Only a moderate difference in probability ratios was observed for RCs, with a probability of 1.06 for providers in financial distress versus 0.85 for financially healthy providers.

**Table 4:** Marginal effects by provider type and provider’s financial health

Provider types	Financial distress	Probability at threshold	Probability directly before threshold	Probability ratio^a^	Ratio of probability ratios^b^
GH + UMC	${\text{NPM}} \ge$ 0 (N = 159,232)	0.094	0.085	1.110***	1.044***
		(0.006)	(0.005)		
	${\text{NPM}} <$ 0 $\left( {{\text{N}} = 23,430} \right)$	0.079	0.069	1.159***	
		(0.011)	(0.009)		
ITC	${\text{NPM}} \ge$ 0 $\left( {{\text{N}} = 42,681} \right)$	0.082	0.055	1.501***	3.202***
		(0.009)	(0.006)		
	${\text{NPM}} <$ 0 $\left( {{\text{N}} = 121,018} \right)$	0.163	0.034	4.806***	
		(0.005)	(0.003)		
RC	${\text{NPM}} \ge$ 0 $\left( {{\text{N}} = 210,636} \right)$	0.080	0.094	0.853***	1.237***
		(0.003)	(0.004)		
	${\text{NPM}} <$ 0 $\left( {{\text{N}} = 418,003} \right)$	0.080	0.076	1.055***	
		(0.002)	(0.002)		

Standard errors in parentheses. N stands for number of observations

*p < 0.1; **p < 0.05; ***p < 0.01

^a^Probability ratios are bootstrapped in order to obtain confidence intervals. *P* values for Wald-test for H_0_: Probability Ratio = $\frac{{{\text{Probability}}\,{\text{at}}\,{\text{threshold}}}}{{{\text{Probability}}\,{\text{directly}}\,{\text{before}}\,{\text{threshold}}}} = 1$

^b^Ratios of probability ratios are bootstrapped in order to obtain confidence intervals. P-values for Wald-test for H_0_: Ratios of Probability Ratio = $\frac{{{\text{Probability }}\,{\text{ratios}}\,{\text{for}}\,{\text{NPM}} < 0}}{{{\text{Probability}}\,{\text{ratios}}\,{\text{for}}\,{\text{NPM}} \ge 0}} = 1$

**Table 5:** Thresholds per subcategory

Subcategory	DRG code	DRG levels	Min hours	Max hours
1. Brain disorder				
	990027160	1	9	50
	990027159	2	51	161
	990027158	3	162	397
	990027157	4	398	Unlimited
2. Organ disorder				
	990027186	1	9	72
	990027185	2	73	232
	990027184	3	233	458 (years 2013—2015)/unlimited (2016-)
	990027183^a^	4	459	Unlimited (years 2013—2015)
3. Musculoskeletal system disorder				
	990027142	1	9	58
	990027141	2	59	128
	990027140	3	129	270 (years 2013—2015)/unlimited (2016-)
	990027139^a^	4	271	Unlimited (years 2013—2015)
4. Nervous system disorder				
	990027168	1	9	67
	990027167	2	68	182
	990027166	3	183	Unlimited
5. Amputation				
	990027151	1	9	70
	990027150	2	71	269
	990027149	3	270	551 (years 2013—2015)/unlimited (2016-)
	990027148^a^	4	552	Unlimited (years 2013—2015)
6. Chronic Pain and mental disorders				
	990027195	1	9	48
	990027194	2	49	130
	990027193	3	131	274 (years 2013—2015)/unlimited (2016-)
	990027192^a^	4	275	Unlimited (years 2013—2015)
7. Paraplegia	990027177	1	9	55
	990027176	2	56	163
	990027175	3	164	354 (years 2013—2015)/unlimited (2016-)
	990027174^a^	4	355	Unlimited (years 2013—2015)

^a^DRG eliminated at the end of 2015

**Table 6:** Summary statistics by subcategory (excluding short rehabilitation (DRG0)

a	1. Brain disorder	2. Organ disorder	3. Musculoskeletal system disorder	4. Nervous system disorder	5. Amputation	6. Chronic pain and mental disorders	7. Paraplegia
Mean weighted hours of treatment	34.53	27.44	25.47	25.74	29.85	31.71	28.21
SD weighted hours of treatment	28.34	19.38	23.24	19.06	22.33	23.79	24.83
Female (%)	40.78	50.38	61.89	46.00	33.16	72.25	37.94
Mean age ( years )	27.93	52.06	43.47	42.88	58.01	41.50	44.89
SD age ( years )	24.55	20.28	20.12	23.31	16.04	15.21	22.26
Presence of other comorbidities (%)	78.48	81.84	39.21	62.07	66.13	69.20	94.91
Mean reimbursement per claim (€)	4235	3465	2865	3277	3683	3529	3903
SD reimbursement per claim (€)	2825	1891	1442	1650	2187	1765	2954
Number of providers (#)	56	55	61	53	42	55	42
Number of providers in financial distress (#)	11	12	18	0	8	14	8
Share of claims from provider in financial distress (%)	22.44	32.84	29.20	0	20.39	33.84	26.62

## Discussion

Previous economic research has shown that abrupt increases in reimbursements may lead to adverse distortions in providers’ behavior. For example, in a stepwise tariff schedule where fees rise abruptly and remain constant until the next such rise in tariffs, some providers may find it advantageous to extend treatments beyond the threshold and end care directly after the threshold. Similarly, a provider might cut the treatment short once a higher tariff has been reached. Such an incentive may be large, as “passing” to the next tariff may lead to an up to fivefold increase in the providers’ marginal revenue per treated individual.^16^ The presence of manipulated discharges has been shown in previous research (e.g. self-employed providers in Dutch mental health care—Douven et al. (2015), private long-term care facilities in the U.S. Medicare-system—Eliason et al., (2018) but in order to understand how financial incentives affect providers’ behavior, we must also identify the exact drivers and influencing factors of this behavior.

In the present research we analyzed treatment durations for medical specialist rehabilitation care in Dutch healthcare facilities for the years 2015 to 2018. Our research shed light on the extent to which an interdisciplinary group of providers (e.g. physicians, physiotherapists, ergotherapists) paid on monthly fixed salaries responded to such financial incentives and how this response differed by provider type. Furthermore, we estimated how financial distress in the organization might have affected the provider’s willingness to manipulate treatment intensity.

Overall, our results suggest no or only mild strategic behavior by three out of four provider types (including general and university hospitals (GH and UMCs), specialized rehabilitation centers (RCs) and other providers (covering mainly multicategorical hospitals), while a strong response was observed for profit-oriented independent treatment centers (ITCs).^17^ In order to test whether providers’ financial health affected the magnitude of the response, we divided providers into two groups based on net profit margins (NPM < 0, NPM ≥ 0). We then compared the probability of manipulated discharges among the two subgroups by provider type. Our results show that the financial distress was associated with marginally higher manipulation probabilities for three of the four provider types. However, it was associated with considerably higher manipulation for ITCs in distress.

This paper utilized a unique dataset of claims-level data provided by a large Dutch health insurer representing 21% of the country’s insured (Vektis Intelligence, 2018). The underlying assumption of our paper is that providers in the Netherlands treat patients independently of where they are insured.^18^ As rehabilitation care is fully covered in the basic package and available to all insured residents, we consider our findings using one insurer to be indicative of the national trends. Our study has shown that abrupt changes in fee-schedules, especially when based on an ‘easy to-follow’ indicator like treatment duration, may lead to distortions in the amount of care that is provided. However, we have also shown that sensitivity to financial incentives is by no means universal among providers. Traditional providers (GH, UMCs, RCs and multicategorical providers) in our dataset show no, or only marginal, responses to incentives, while ITCs demonstrated much stronger effects.

As the methods used in the paper focused on one threshold and mainly on two time periods in the treatment process (the period at the threshold and directly before the threshold), it did not allow us to test the exact share of the distortions caused by overtreatment (a deliberate extension of treatment durations) versus undertreatment (a deliberate early discharge of patients before the optimal point). However, it is safe to assume that, at least, some of the patients received more care than what they would have received under a smoother reimbursement schedule. These extra treatment hours may have positive, but likely minimal, benefits for these patients. Unfortunately, our dataset does not allow us to estimate extent of such additional benefits.^19^

The distribution of the normalized index of treatment times in Fig. 1 indicates monotone decreasing and convex curves for three of the four graphs with the sole exception of ITCs and only at the discontinuity in the reimbursement schedule. This suggests that the counterfactual curve, the distribution of treatment times in a hypothetical world without discontinuities in financial incentives, would also be smooth and decreasing and that the spike seen at the threshold for ITCs is, in fact, financially motivated. Nonetheless, although it is unlikely to be the case, we cannot completely rule out the possibility that this spike is part of the natural process of treatment and not a result of a change in financial incentives.

Although our results seem to indicate a strong relationship between the financial health of the organization and strategic discharge behavior for ITCs, this relationship could be confounded by financial accounting choices. It is possible that the negative net profit margin observed in our dataset is not a result of low revenues, but caused by other factors that could motivate management to accept losses. Further research (e.g. using different indicators of financial health or observing newly merged entities) could strengthen the evidence of this relationship.

As a consequence of our analysis one might attempt to find an optimal reimbursement schedule leading to the least amount of distortions. However, such schedule is difficult to find: on the one hand, a smooth curve could be obtained by paying providers per treatment hour of care, an equivalent of a fee-for-service (FFS) reimbursement design. On the other hand, the payer may decide to pay a flat fee for all care independent of treatment duration. In fact, both of these cases would lead to distortions of their own kind. The former could bring about longer treatment times (as shown by Coulam & Gaumer, 1992, Ellis & McGuire, 1986), while the latter may lead to patient-selection towards “lighter” patients requiring less care (McGuire, 2000). A combination of the two payment schemes as recommended by Ellis and McGuire (1986) would certainly diminish the behavioral responses at tariff thresholds.

However, rather than searching for the optimal reimbursement design, the regulator may find it more valuable to find factors that drive providers to alter the course of treatment as a response to financial incentives. Based on our results in Analysis I, one might conclude that profit-orientation alone can lead to strong response by a provider. However, in Analysis II we show that manipulation among financially healthy ITCs is only moderately larger than for traditional providers, indicating that it is the financial health of the organization rather than solely its profit orientation that drives this behavior. This latter result suggests a new explanation for the positive findings in providers’ response to financial incentives not addressed by economic literature in the past. Furthermore, it suggests that DRG systems, which were intended to lead to more efficient provision of care, when coupled with poor financial performance of the provider may lead to large inefficiencies.

**Table 7:** Probit results by provider type

	Dependent variable:			
	Closed			
	GH + UMC	ITC	RC	Other
Time	2.179***	− 0.506***	0.678***	0.581***
	(0.031)	(0.024)	(0.009)	(0.030)
Time2	− 1.015***	0.228***	− 0.237***	− 0.230***
	(0.021)	(0.010)	(0.004)	(0.014)
t_before	− 0.449***	− 0.376***	− 0.158***	− 0.286***
	(0.028)	(0.029)	(0.009)	(0.032)
t_at	− 0.394***	0.368***	− 0.127***	− 0.102***
	(0.031)	(0.020)	(0.009)	(0.030)
Constant	− 1.561***	− 1.203***	− 1.479***	− 1.416***
	(0.008)	(0.010)	(0.003)	(0.011)
Observations	209,952	163,699	1,248,089	108,940
Log likelihood	− 88,541.520	− 48,009.120	− 420,928.900	− 37,314.820
Akaike Inf. Crit	177,093.000	96,028.250	841,867.800	74,639.630

*p < 0.1; **p < 0.05; ***p < 0.01

**Table 8:** Marginal effects results by provider type

	Dependent variable:			
	Closed			
	GH + UMC	ITC	RC	Other
Time	0.507***	− 0.079***	0.123***	0.108***
	(0.007)	(0.004)	(0.002)	(0.006)
Time2	− 0.236***	0.035***	− 0.043***	− 0.043***
	(0.005)	(0.002)	(0.001)	(0.003)
t_before	− 0.082***	− 0.046***	− 0.026***	− 0.045***
	(0.004)	(0.003)	(0.001)	(0.004)
t_at	− 0.074***	0.071***	− 0.021***	− 0.018***
	(0.004)	(0.005)	(0.001)	(0.005)
Observations	209,952	163,699	1,248,089	108,940
Log likelihood	− 88,541.520	− 48,009.120	− 420,928.900	− 37,314.820
Akaike Inf. Crit	177,093.000	96,028.250	841,867.800	74,639.630

*p < 0.1; **p < 0.05; ***p < 0.01

**Table 9:** Probit results by provider type and provider’s financial health

	Dependent variable					
	Closed					
	GH + UMC		ITC		RC	
	(${\text{NPM}} >$ 0)	(${\text{NPM}} \le$ 0)	(${\text{NPM}} >$ 0)	(${\text{NPM}} \le$ 0)	(${\text{NPM}} >$ 0)	(${\text{NPM}} \le$ 0)
Time	2.966***	1.632***	2.604***	− 0.838***	0.543***	0.548***
	(0.045)	(0.093)	(0.082)	(0.029)	(0.020)	(0.013)
Time2	− 1.569***	− 0.713***	− 1.340***	0.396***	− 0.204***	− 0.166***
	(0.038)	(0.065)	(0.065)	(0.011)	(0.009)	(0.005)
t_before	− 0.410***	− 0.442***	− 0.590***	− 0.339***	− 0.080***	− 0.121***
	(0.033)	(0.069)	(0.058)	(0.036)	(0.021)	(0.014)
t_at	− 0.345***	− 0.357***	− 0.363***	0.579***	− 0.170***	− 0.090***
	(0.039)	(0.073)	(0.059)	(0.022)	(0.023)	(0.015)
Constant	− 1.730***	− 1.525***	− 1.748***	− 1.282***	− 1.418***	− 1.533***
	(0.011)	(0.025)	(0.021)	(0.013)	(0.008)	(0.006)
Observations	159,232	23,430	42,681	121,018	210,636	418,003
Log likelihood	− 66,650.960	− 9,373.831	− 17,020.380	− 28,610.450	− 71,499.240	− 129,479.500
Akaike Inf. Crit	133,311.900	18,757.660	34,050.760	57,230.910	143,008.500	258,969.000

*p < 0.1; **p < 0.05; ***p < 0.01

**Table 10:** Marginal effects results by provider type and provider’s financial health

	Dependent variable					
	Closed					
	GH + UMC		ITC		RC	
	(${\text{NPM}} >$ 0)	(${\text{NPM}} \le$ 0)	(${\text{NPM}} >$ 0)	(${\text{NPM}} \le$ 0)	(${\text{NPM}} >$ 0)	(${\text{NPM}} \le$ 0)
Time	0.685***	0.358***	0.568***	− 0.101***	0.100***	0.091***
	(0.010)	(0.020)	(0.018)	(0.003)	(0.004)	(0.002)
Time2	− 0.362***	− 0.156***	− 0.292***	0.048***	− 0.037***	− 0.028***
	(0.009)	(0.014)	(0.014)	(0.001)	(0.002)	(0.001)
t_before	− 0.076***	− 0.076***	− 0.092***	− 0.032***	− 0.014***	− 0.019***
	(0.005)	(0.009)	(0.006)	(0.003)	(0.004)	(0.002)
t_at	− 0.066***	− 0.064***	− 0.064***	0.102***	− 0.028***	− 0.014***
	(0.006)	(0.010)	(0.008)	(0.005)	(0.003)	(0.002)
Observations	159,232	23,430	42,681	121,018	210,636	418,003
Log likelihood	− 66,650.960	− 9,373.831	− 17,020.380	− 28,610.450	− 71,499.240	− 129,479.500
Akaike Inf. Crit	133,311.900	18,757.660	34,050.760	57,230.910	143,008.500	258,969.000

*p < 0.1; **p < 0.05; ***p < 0.01

## Appendix

See Figs. 2, 3 and Tables 5, 6, 7, 8, 9 and 10.

## Footnotes

1 Upcoding is when a provider fraudulently changes the medical records of the patient in order to increase reimbursements.

2 The distinction between upcoding and treatment manipulation (also referred to in the paper as strategic discharge) must be noted. While upcoding is a criminal act, treatment manipulation is not criminal only unethical behavior by the provider.

3 ITCs were initially introduced during the health care reform of 2005 to ease the heavy burden of waiting lists, but these small private for-profit health care facilities quickly became an integral part of the new liberalized healthcare system performing high-volume and routine medical procedures. ITCs tend to focus on one specialty and, due to their limited scope and high volume, they can often undercut regular hospitals in terms of price. The number of ITCs has grown exponentially since their introduction: from 120 head offices in 2007 to 173 registered in 2010 and 229 in 2016.

4 We categorize these claims as “Other” providers.

5 Specialist care following an emergency room visit is an exception to this rule. In this case, no GP referral is required [12].

6 Although the provider is given 9 weighted hours to establish a diagnosis, in the majority of the cases this period is considerably shorter (on average 1.4 h in our dataset).

7 In addition to the rehabilitation care described in this paper, there is another segment of care referred to as geriatric rehabilitation care, provided in nursing homes under the supervision of a geriatric specialist [11]. This type of care is meant for frail elderly persons where the aim of the treatment is no longer a full recovery to active life. Some patients that begin their treatments in medical rehabilitation care may be quickly transferred to geriatric rehabilitation institutions. These transfers occur in the first part of patient-provider encounters.

8 It is important to emphasize that rehabilitation care requires a large medical staff of physicians, physiotherapists, ergo- and speech therapists, etc., therefore these institutions are typically not stand-alone facilities, but larger organizations with many employees. Additionally, our dataset only includes rehabilitation providers that provide contracted (in-network) care. Due to the administrative costs associated with negotiating a contract with the payer, these organizations tend to be larger with more than one location. It is still possible that some ITCs in our dataset are, at least in part, owned by one or more employees, and this might elicit a more profit-maximizing behavior by these employees even if the individual is paid using a monthly salary. However, this is unlikely to have a large effect on our results.

9 Algemeen Gegevens Beheer-codes or AGB-codes in Dutch.

10 NPMs were obtained from the Dutch Chamber of Commerce from publicly available annual financial accounts for all healthcare organizations. In certain cases, more than one organization was registered under the same Chamber of Commerce code (KvK-code). We categorized these organizations as being in financial distress if at least one of the organizations had a negative NPM.

11 Some provider could not be linked with KvK-codes. Claims registered by these providers were excluded from Analysis II. See Table 2 for number of providers and percentage of claims included in Analysis II by provider type.

12 Combining subcategories per provider meant that we needed to normalize treatment times, since the same provider may treat a combination of conditions (e.g. chronic pain with the first threshold at 49 weighted hours and musculoskeletal system disorder with the first threshold at 59 h). This led to varying thresholds and ranges of treatment times for the same provider. In order to solve this problem, we used the following formula to center and normalize treatment times around the first threshold:

$$Norm. Index= \frac{(WHR-T1)}{T1}$$

We presented the distribution of the obtained normalized index by provider type in Fig. 1.

13 Using a normalized index allowed the aggregation of claims by provider type, but it also meant that the natural period of observation (i.e. treatment hour) was lost and the bin size to break the normalized treatment index into time periods had to be chosen manually. As with a histogram, using excessively wide bins may average out data, hiding important trends, whereas using excessively narrow bins may lead to unnecessary noise. Using visual analysis we concluded that 0.05 was the most appropriate bin size for representing our distribution (as in Fig. 1). This bin size led to a smooth curve without random fluctuations, but also with a visible trend line at the threshold.

14 This is a reasonable assumption given that three of the four curves (with the exception of ITCs) in Fig. 1 demonstrate monotone decreasing and convex curves.

15 The probability ratio captures the difference in the probability of discharge at $t_{before}$ compared to the probability of discharge at $t_{at}$ i.e. the marginal effect of moving from $t_{before}$ to $t_{at}$. Average marginal effects, commonly calculated following probit models are not applicable for our model, as this would compare the probability of discharge at $t_{at}$ (and at $t_{before}$) with the average overall probability of discharge.

16 In 2015, the average tariff received for up to 50 weighted hours of treatment was €2711. By extending treatment by one hour, the tariff increases to €13,905.

17 Our results likely underestimate the overall magnitude of the problem, as our dataset is limited to ‘in-network’ ITCs. Dutch health insurers are required by law to reimburse up to 70% of costs incurred at ‘out of network’ providers (mostly care provided by ITCs), but the detailed activities for such care are not readily available to the health insurer. While we have no way of testing whether treatment manipulation is similarly prevalent among ‘out-of-network’ providers, we also have no reason to assume that they would respond any differently facing equivalent incentives as their ‘in-network’ counterparts.

18 As far as we know this has never been scientifically proven, but it was also confirmed by medical professionals we interviewed for this paper.

19 Marginal benefits of treatment beyond the optimal treatment level may, in certain cases, be negative due to the additional risks involved with treatment (e.g. in the case of surgical procedures). However, as our paper focused on rehabilitation care in outpatient facilities, we can safely assume that the risks associated with treatment are negligible. Hence, the marginal benefit of additional treatment is >  = 0.
